# Dynamic Response and Numerical Simulation of Closed-Cell Al Foams

**DOI:** 10.3390/ma15228207

**Published:** 2022-11-18

**Authors:** Yinzheng Xia, Jianchao Shi, Yongliang Mu

**Affiliations:** 1 School of Metallurgy, Northeastern University, Shenyang 110819, China; 2 Guizhou Aerospace Tianma Electromechanical & Technology Co., Ltd., Zunyi 563000, China

**Keywords:** cellular materials, drop hammer impact, dynamic response, energy absorption, finite element analysis

## Abstract

The drop hammer impact test was carried out to investigate the dynamic response of closed-cell Al foams. A relatively reasonable method was also developed to evaluate the velocity sensitivity of cellular material. The typical impact load–displacement curve exhibited two stages containing the initial compression stage and the progressive crushing stage. Three compressive damage behaviors and four failure modes of closed-cell Al foams were revealed, while the effect of velocity on the impact properties and the energy absorption capacity of different specimens were investigated. The results showed that the specific energy absorption of the specimens increased with the increasing density of the specimen and the impact velocity. However, the specimens with higher specific energy absorption seemed not to indicate better cushioning performance due to the shorter crushing displacement. In addition, the uniaxial impact simulation of two-dimensional (2D) Voronoi-based foam specimens was conducted at higher impact velocities. The simulation results of impact properties and deformation behavior agreed reasonably well with the experimental results, exhibiting similar velocity insensitivity of peak loads and deformation morphologies during uniaxial impact.

## 1. Introduction

Metal foams attract growing attention due to the heterogeneous and discontinuous porous structure [1,2,3,4]. The plastic plateau in the stress–strain response of the metal foam resulted from the special pore structure is the principal reason for the excellent cushioning energy absorption characteristics of the metal foams [5,6,7,8]. Metal foams have been widely used in various applications such as transportation, aerospace, and defense industries, challenged by different service environments.

The research aiming at deformation behavior and mechanical properties under quasi-static conditions is relatively well established. Almost all the results conclude that quasi-static response goes through three stages containing the linear elastic stage, the plastic plateau stage, and the densification stage. Gibson and Ashby [9] first developed the most representative prismatic model, giving the quantitative relationship between density and cellular structure strength. On the basis of the prismatic model, researchers have systematically investigated the effects of a cell’s size, shape, anisotropy, cell wall thickness, and related factors on the mechanical properties of the material [10,11,12,13,14,15]. In addition, the deformation mechanism and deformation process of closed-cell aluminum foam during quasi-static compression were also revealed [16]. Four failure modes at the cell/membrane level including bending and plastic hinges, ductile tearing, fracture, and stretching were characterized, which corresponded to diverse energy absorption mechanisms. 

In recent years, many attempts have been made to study the dynamic response of metal foams [17,18,19,20]. Stress enhancement is a significant characteristic during dynamic response. The micro-inertia effect has been proposed to explain stress enhancement under impact by many researchers [21,22]. The mechanical responses of circular aluminum extrusions filled with aluminum foams under different strain rates were investigated [23]. The result showed that the mean load under dynamic loading conditions was higher than the static case, which was due to the inertia effects arising in the extrusion walls during crushing. The stress enhancement was usually considered for impact velocity greater than 45 m/s. The deformation mode and energy absorption characteristics under dynamic impact were also evaluated. Ramachandra [24] reported that the absorbed energy increased marginally with the velocity within the quasi-static regime, but increased significantly at velocities greater than 10 m/s. The appearance of shockwave effects may be responsible for the diverse phenomenon. Tan [25] explored the relationship between the deformation mode of aluminum foam and impact velocity. For static compression as well as sub-critical impact, the aluminum foam deformed through the cumulative multiplication of discrete crush bands. The impact surface is crushed first and propagated progressively inward at super-critical impact velocity. Moreover, several reinforcing materials such as expanded perlite, ceramic spheres, nanotubes, and nanowhiskers have been added to aluminum foam matrix nowadays, and the dynamic response of metal matrix syntactic foams (MMSF) was investigated by a number of workers [26,27,28,29]. The micro-inertial effect also affects the rate sensitivity of the MMSFs [30]. However, there are still no definite conclusions on whether the MMSF itself is sensitive to strain rates [30,31]. Yang [26] reported the same shear deformation mode of the MMSFs at different strain rates, which was different from the test results of metal foams. In summary, the dynamic responses of metal foams and MMSFs still demand further exploration.

However, it is hard to appreciate the intermediate states of collapse and the evolution of the in situ collapse through post-impact observation of specimens due to the instantaneous deformation process during dynamic impact. Thus, more investigations based on the finite element analysis aiming at the deformation behavior of metal foams were performed. Different strain rate conditions comprising quasi-static [32,33] and dynamic [34,35] were imposed on various foam models. A number of researchers have employed multifarious models to reproduce the real foam structure and deformation behavior as much as possible, e.g., homogeneous foam structure model [36,37], X-ray micro-computed tomography (XCT) reconstructed foam geometry [38,39,40], random Voronoi foam [41,42], etc. Among these modeling strategies, the meshing of complex models and the selection of materials’ constitutive relationships are still urgent problems to be solved.

In the present study, we expect to develop a relatively reasonable method to evaluate the reliability and reproducibility of uniaxial impact behavior for closed-cell aluminum foams. Thus, the main objective of this paper is not only to obtain specific impact mechanical property parameters, but more importantly to provide an analytical method for the dynamic response of the cellular structure. 

Consequently, the drop hammer impact test was carried out to investigate the dynamic response of the closed-cell Al foams under different impact velocities. Additionally, a constant initial impact energy was subjected to the impact system and the initial velocity of the hammer was adjusted by varying the mass of the hammer. The impact performance and energy absorption capacity of Al foams at different impact velocities were characterized. Meanwhile, two-dimensional (2D) Voronoi models were built to replicate the deformation of closed-cell aluminum foam at higher velocities by finite element simulation.

## 2. Materials and Methods

### 2.1. Materials and Specimens

**Figure 1:**
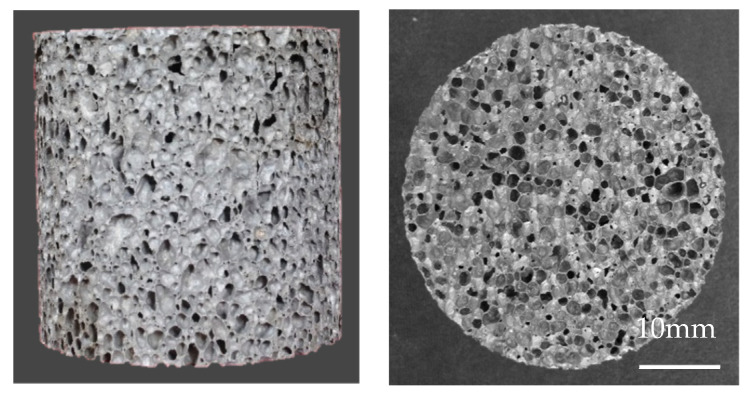
The closed-cell Al foam specimen for impact testing.

The closed-cell aluminum foams studied in present work were prepared from melt foaming method. Pure Al (Fushun Aluminum Co., Ltd., Fushun, China, 99.9%) was selected as the raw material for the experimental specimens. The manufacturing method involved the following steps: (1) the pure Al and Ca (Sichuan Jianzhong Company, Chengdu, China, 98.0%) were melted in crucible furnace; (2) the TiH_2_ (Fushun Aluminum Co., Ltd.) foaming agent was added into the melt; (3) after holding for a certain period, the crucible was taken out for the furnace; (4) the melt was cooled to room temperature in air. Further details about the fabrication process were described in ref. [43]. All the specimens were ground into cylinders with dimensions of *Φ* 40 × 50 mm and the density of the specimens ranged from 0.267 g/cm^3^ to 0.657 g/cm^3^. Figure 1 depicts the morphology of impact test specimen.

### 2.2. Testing Method

The uniaxial impact tests (ASTM D2444) were carried out with Instron9250HV drop hammer system (INSTRON, Canton, OH, USA). The impact test system was equipped with a drop tower testing machine, a system for measuring momentary pressure force, and a system for acquiring data. Additionally, the instantaneous crushing distance was recorded using a laser-grating-photoelectron system. An accelerometer was mounted on the upper bottom of the hammer to measure the momentary acceleration. 

In the present study, all specimens were subjected to 246.84 ± 3.27 J initial kinetic energy to investigate the dynamic impact velocity sensitivity of the closed-cell Al foam. Impact tests of different velocities of 3 m/s, 5 m/s and 7.8 m/s were conducted, corresponding to the various hammer mass of 55.64 Kg, 19.68 Kg, and 7.98 Kg. The kinetic energy values in this test were determined after extensive experiments to ensure that the specimens could absorb all the energy.

The quasi-static compression tests (ASTM E9) were performed using CMT5105 Universal Testing Machine (MTS, Eden Prairie, MN, USA) with a constant compression velocity of 2 mm/min.

### 2.3. Experimental Principles and Data Processing

The momentary impact load can be expressed as [17]

(1) P(t)=M[g+a(t)]

where *P(t)* is the momentary compression load, *M* is the tup mass, *g* is the acceleration of gravity, and *a(t)* is the measured momentary acceleration.

The corresponding momentary velocity and displacement can be calculated by integrating the acceleration once and twice, respectively.

(2) $$v\left(t\right)=v\left(0\right)+\int_{0}^{t}a\left(\tau \right)d\tau$$

(3) $$s\left(t\right)=\int_{0}^{\tau }d\tau \int_{0}^{t}a\left(t\right)dt-s\left(0\right)$$

where *v*(0) and *s*(0) are initial impact velocity and initial compression displacement separately. The load–displacement relationship *P*(*s*) can be achieved by combining Equations (1) and (3). Integrating *P*(*s*) yields the impact energy absorbed by the specimens.

(4) $$E=\int_{0}^{\Delta L}P\left(s\right)ds$$

The impact energy delivered to the specimens is

(5) $$E^{\prime }=Mg\left(H+\Delta L\right)$$

where *H* is the distance between the bottom surface of the hammer and the upper surface of the specimens, Δ*L* is the crushed length of the specimens. Among all the impact tests, |*E’ − E/E’*| < 5%, verifying the excellent accuracy of the impact testing system.

The instantaneous specific load can be obtained by instantaneous loads divided by cross-section areas and densities of specimens, for eliminating the effect of size and density on the analysis. Processing the data with this simplified normalization method is a preliminary attempt with some errors.

(6) $$F_{s}\left(t\right)=P\left(t\right)/A\rho$$

where *A* and *ρ* are the cross-section area and average density of the specimens.

In regard to the energy absorption ability, a significant parameter is specific energy absorption, established as the energy dissipated per unit sample weight.

(7) $$E_{s}=E/A\rho \Delta L$$

when the specific load is expressed as a function of displacement,

(8) $$E_{s}=\frac{1}{\Delta L}\int_{0}^{\Delta L}F_{s}\left(s\right)ds$$

### 2.4. Finite Element Models

In this work, the uniaxial impact tests at higher velocities (higher than 7.8 m/s) were not performed due to the limitation of the experimental device. In addition, it was hard to appreciate the intermediate states of collapse and the evolution of the in situ collapse through post impact observation of specimens. In order to explore the complete deformation behavior of the specimens at greater impact velocities, the uniaxial impact simulation based on the finite element analysis was carried out. The simulation results will be contrasted with the experimental results for analysis.

**Figure 2:**
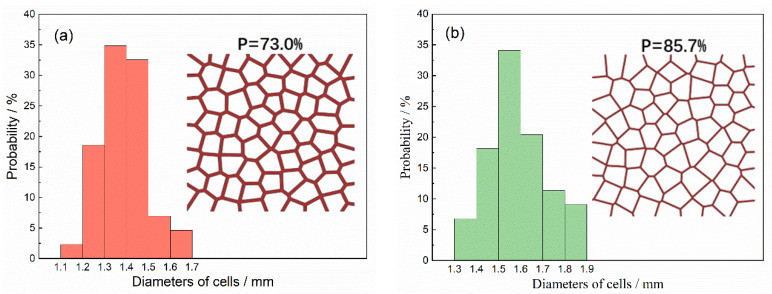
Two-dimensional Voronoi foam specimens with different porosities and their cell size distributions (**a**) 73.0%, (**b**) 85.7%.

The 2D Voronoi structure was employed to generate the closed-cell aluminum foam. To eliminate the size effect [44], the transverse and longitudinal direction of the foam model shall include at least seven cells. The closed-cell foam specimen with 73.0% (0.729 g/cm^3^) and 85.7% (0.386 g/cm^3^) porosity was constructed in a size of 10 × 10 mm with about 80 nuclei, as shown in Figure 2. The porosity of the specimens was altered by changing the cell walls’ thickness. Figure 2 also presents the cell size distributions of 2D Voronoi foam specimens. The cell size was the equivalent diameter obtained by counting the area of the cell.

**Figure 3:**
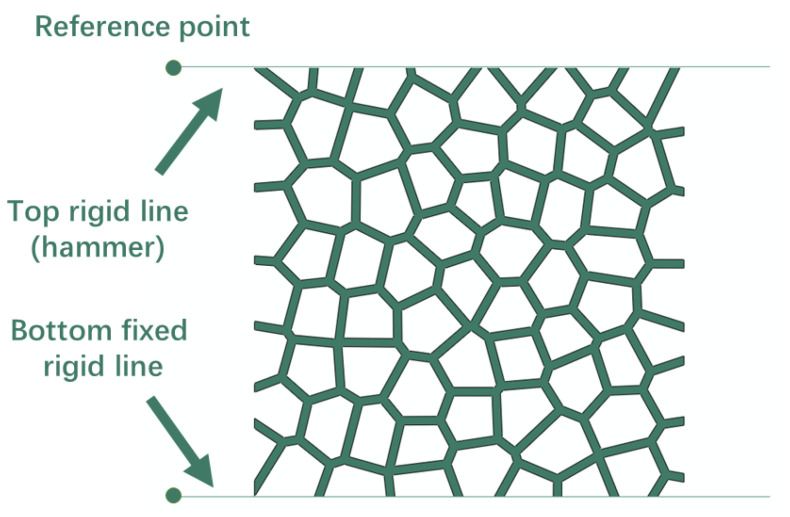
The schematic of simulation conditions.

**Figure 4:**
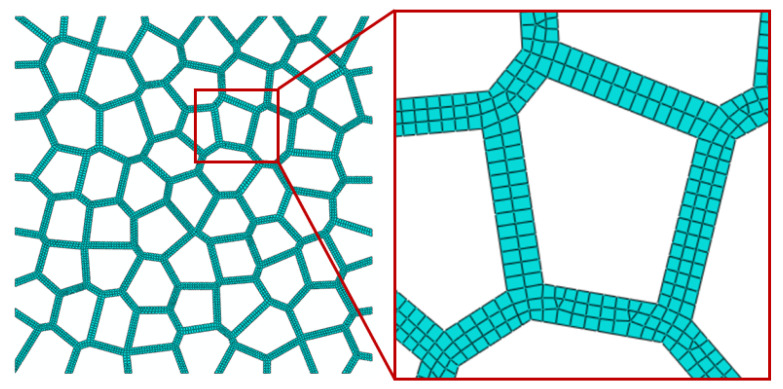
Models with 73.0% porosity corresponded to element sizes of 0.07 mm after meshing.

The matrix material is assumed to be linear strain hardening material with density *ρ* = 2700 kg/m^3^, Young’s modulus *E_s_* = 70,000 MPa, Poisson’s ration *υ_s_* = 0.33, yield strength *σ_ys_* = 110 MPa and tensile strength *σ_ts_* = 160 MPa. As Figure 3 depicts, two discrete rigid lines were built and assigned on the top and bottom of 2D Voronoi foam specimens, with reference points on each left end. The top rigid line functioned as a drop hammer during impact simulation, assigning the velocity and mass through the reference point, while the bottom rigid line was used as a fixed platform to carry the specimen. Contact between the foam specimen and the rigid line and that between all possible surfaces within the foam specimen were considered with a friction coefficient of 0.2. The 2D Voronoi-based foam model was meshed by using S4R, with the Medial axis algorithm and the element shape of Quad-dominated. The characteristic lengths of element size of the models with 73.0% and 85.7% porosity were set to approximately 0.07 mm and 0.02 mm, respectively, through a mesh sensitivity study. Figure 4 shows the schematics of the model with 73.0% porosities after meshing and the enlarged local meshing details. 

The numerical uniaxial impact behavior of the closed-cell Al foam was also investigated using a research methodology identical to the impact experiment. Specimens with porosities of 73.0% (0.729 g/cm^3^) and 85.7% (0.386 g/cm^3^) were subjected to the constant initial kinetic energies of 0.6 J and 0.15 J, respectively. The initial impact energy for different foam specimens was also determined through multiple impact simulations to satisfy the energy absorption requirements according to Equations (4) and (5). The variation in initial kinetic energy between experiments and simulations was attributed to the size and two-dimensional nature of the specimens during the impact simulation. Uniaxial impact simulations of different velocities of 10 m/s, 20 m/s, and 30 m/s were carried out with the FE code Abaqus/Explicit. The analysis step times of the models with impact velocities of 10 m/s, 20 m/s, and 30 m/s were 0.0010 s, 0.0007 s, and 0.0004 s, respectively, to ensure sufficient time for the complete impact simulation process. The specific parameter settings for the impact simulation were listed in Table 1. The load–displacement curves of the top rigid line (hammer) will be used in the analysis of the simulation results for the 2D Voronoi-based foam specimens. In addition, this simulation study was particularly aimed at the deformation process of the specimens at higher impact velocities.

## 3. Results and Discussion

### 3.1. Uniaxial Impact Test Curves

**Figure 5:**
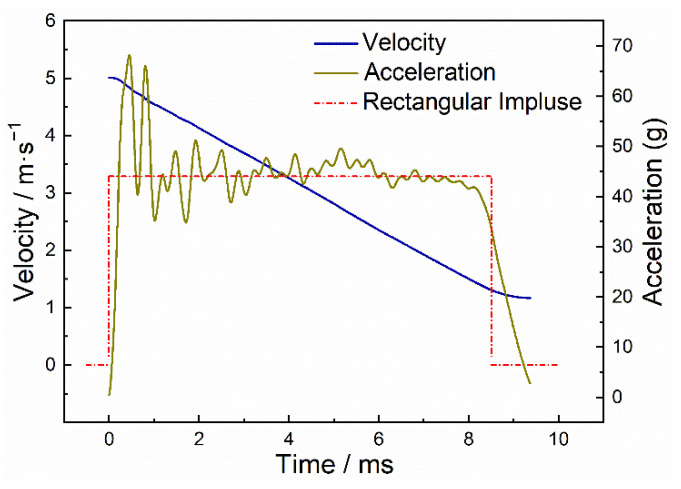
Typical acceleration and velocity curves for closed-cell Al foam.

**Figure 6:**
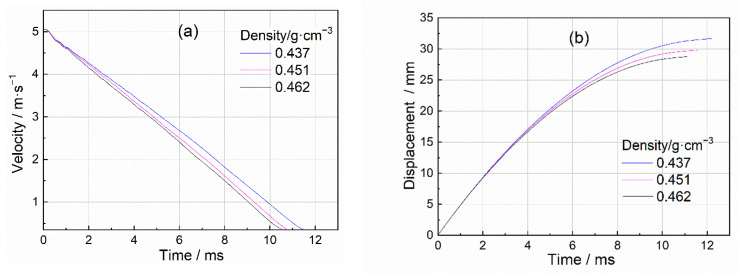
Velocity and displacement duration of Al foam with different porosities (**a**) velocity duration (**b**) displacement duration.

Figure 5 and Figure 6 depict the typical acceleration, velocity, and displacement curves by processing the test data by the method recorded in Section 2.3. The acceleration curve approximated a rectangular pulse, causing the impact velocity to decrease almost linearly from the initial contact velocity to zero. Figure 6 shows the dynamic velocity and displacement history of closed-cell Al foam with different densities at the same initial impact energy and velocity (5 m/s). All data processing and analysis in the following were based on Figure 6.

**Figure 7:**
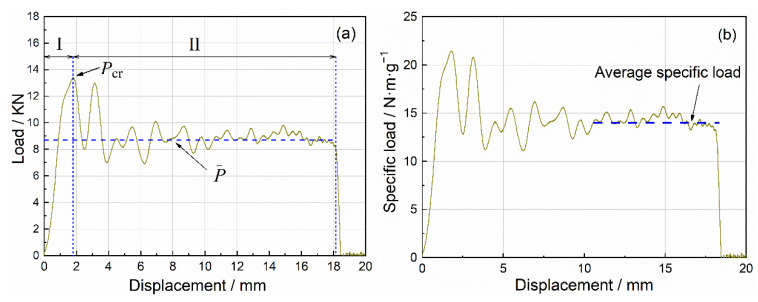
Typical impact curve for closed-cell Al foam (**a**) load–displacement curve (**b**) specific load–displacement curve.

**Figure 8:**
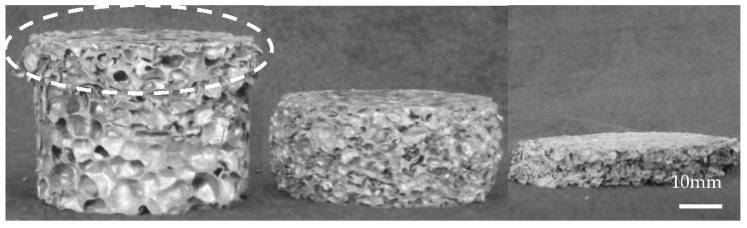
Deformation process of closed-cell Al foam with the density of 0.451 g/cm^3^ under uniaxial impact velocities of 5 m/s.

Figure 7 displays the typical load–displacement curve and specific load–displacement curve of closed-cell Al foam specimens under uniaxial impact compression. Most of the test data in this study exhibited a similar pattern. Figure 8 presents the corresponding deformation process, which could be roughly divided into two stages containing the initial compression stage and the progressive crushing stage. Stage I shown in Figure 7 was the initial compression stage, i.e., the elastic compression stage, where the load increased almost linearly with displacement. The specimens underwent local buckling and the compression load decreased as the load reached *P*_cr_. At stage II, the progressive crushing stage, localized larger-scale damage appeared within the specimens. Additionally, the impact load oscillated along a horizontal line (the average load *P*), companied by the little varied compression load. Higher average load *P* and longer compression stroke during the progressive crushing stage indicated a stronger energy absorption of the specimens. In the following, we applied the specific load curve instead of the load curve unless otherwise specified to eliminate the influence of materials and structures.

### 3.2. Compression Damage Behaviors

**Figure 9:**
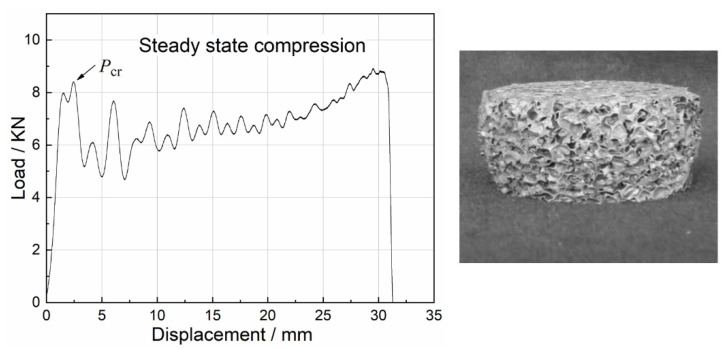
Load–displacement curve for steady state compression and damaged specimen.

**Figure 10:**
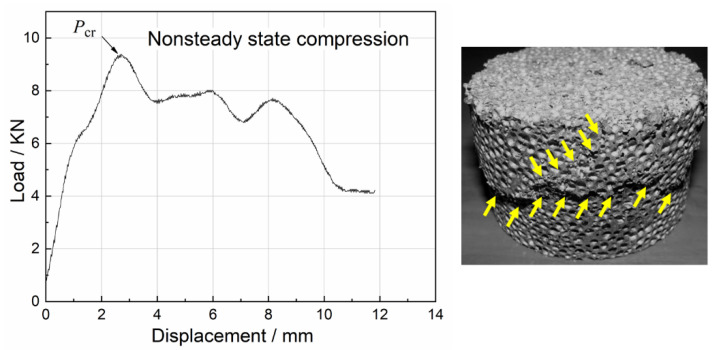
Load–displacement curve for nonsteady state compression and damaged.

**Figure 11:**
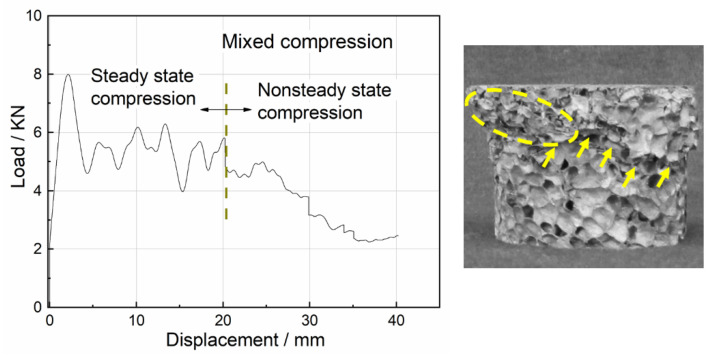
Load–displacement curve for mixed compression and damaged specimen.

Uniaxial impact test results of all foam specimens revealed three typical damage modes: steady state compression, nonsteady state compression and mixed compression. The load–displacement curves for the three damage modes and the corresponding damaged specimens are depicted in Figure 9, Figure 10 and Figure 11.

During steady-state compression, the specimen primarily suffered local damage, followed by successive crushing with further loading. As a result, the specimen underwent global plastic deformation. In the next stage, the compressive load was approximately sawtooth-shaped fluctuations and increased slightly as shown in Figure 9. The compression behavior of most of the foam specimens revealed steady-state compression, which was also the main subject of this study.

The specimens suddenly occurred in the whole damage and lost load-bearing capacity under the action of compression load. The compression load increased to *P*_cr_ and then rapidly decreased, i.e., nonsteady state compression. The typical load–displacement curve of nonsteady state compression and the related damaged specimen are given in Figure 10. During nonsteady state compression, the specimens generated fewer cracks with larger residual fragments, resulting in less absorbed energy. It was found that the specimens with higher density were more likely to exhibit nonsteady state compression.

Figure 11 displays the typical load–displacement curve for mixed compression and the corresponding damaged specimen. After the initial stage of plastic deformation, the accumulation of several defects within the specimen such as a large deformation slip band gradually became obvious and achieved dominance. The subsequent compression stage was unable to maintain the progressive damage and the load-bearing capacity degraded rapidly. It was evident that the energy absorption of the mixed compression lay between the steady state compression and the nonsteady state compression. In addition, the mixed compression was more commonly observed in the specimens with lower densities.

### 3.3. Energy Absorption Analysis

**Figure 12:**
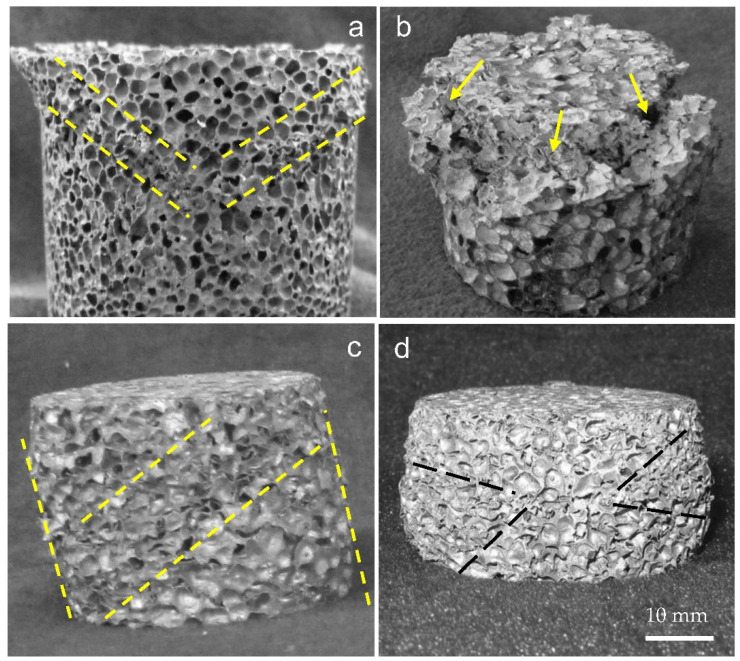
Uniaxial impact failure modes (**a**) “V”-shaped deformation mode (**b**) end crack damage (**c**) shear failure mode (**d**) localized buckling failure mode.

Figure 12 shows four various failure modes of closed-cell aluminum foam specimens during uniaxial impact testing: (a) “V”-shaped deformation mode; (b) end crack damage mode; (c) shear failure mode; (d) localized buckling failure mode. The different failure modes of the specimens during compression represented separate energy absorption mechanisms. In addition, the specimens mostly presented multi-mode mixed deformation in the impact process, which was responsible for the complexity of the foam structure. 

It can be found from Figure 6 that the crushing duration of the specimens depended on the densities of the specimens. The results revealed that the specimen with higher density had a shorter crushing duration and higher peak load when subjected to impact load (5 m/s). The shorter crushing duration indicated the smaller final crushing displacement of the specimen. It was clear from Equations (7) and (8) that, with the constant impact energy input, even though the specific energy absorption of the high-density specimen was similar or much higher than low-density specimen, the cushioning performance will not be ideal due to the small crush displacement.

**Figure 13:**
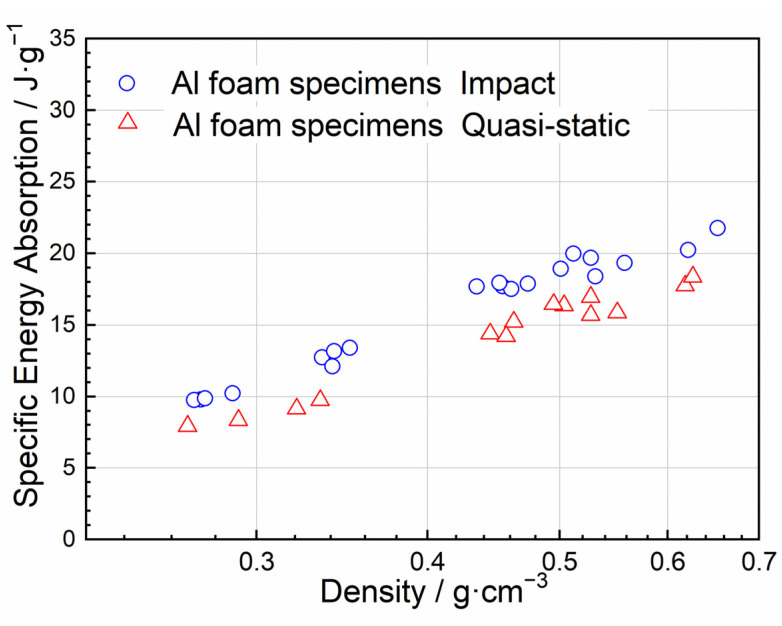
Comparison of dynamic specific energy absorption with that of quasi-static.

The quasi-static compression strain energy density of the closed-cell aluminum foam specimen was converted to the specific energy absorption under quasi-static compression by dividing the apparent density of the specimen. Since the crushing length of the uniaxial impact specimen was between 32–40 mm, which was comparable to the quasi-static compression strain of about 0.7. Thus, the specific energy absorption at the quasi-static compression strain of 0.7 and the impact velocity of 5 m/s is plotted in Figure 13. It can be observed from Figure 13 that the dynamic specific energy absorption value was slightly higher than the quasi-static value. The strain rate effect and inertia effect of the material under dynamic loading conditions increased the value of the load, resulting in more impact energy being absorbed by the closed-cell aluminum foam for the same deformation displacement. Combined with the analysis above, the high specific energy absorption value did not indicate good cushioning performance. Therefore, materials with suitable specific energy absorption should be reasonably selected when designing the energy absorption structure.

### 3.4. Impact Performance’s Velocity Sensitivity

**Figure 14:**
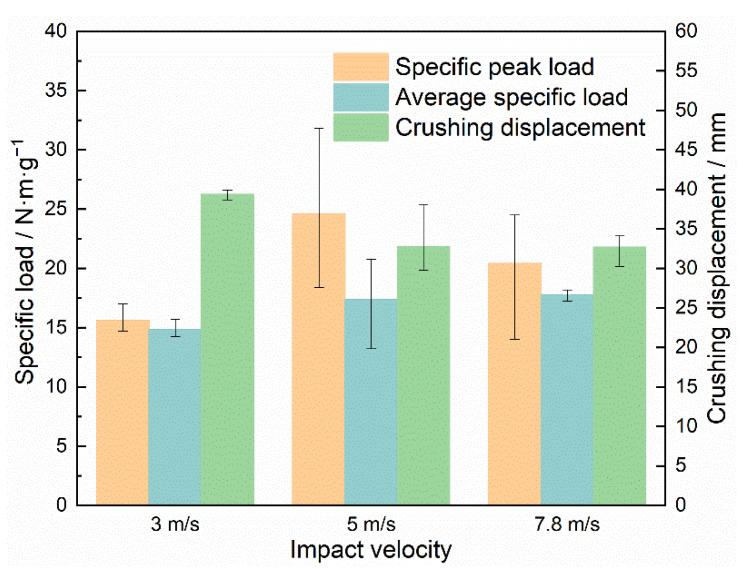
Specific peak loads, average specific loads, and crushing displacements of specimens under various impact velocities.

**Figure 15:**
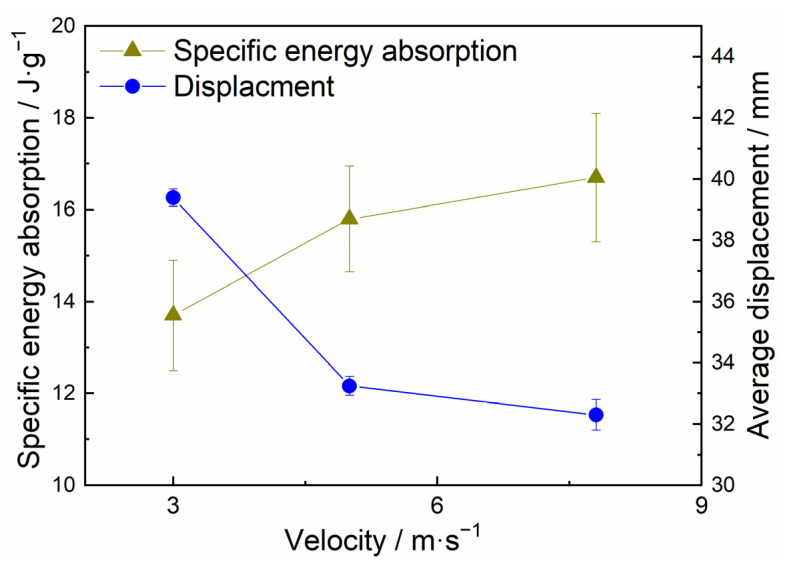
Velocity sensitivity of Al foam in axial impact.

Figure 14 statistically presents the specific peak loads, average specific loads, and crushing displacements of the closed-cell aluminum foam specimens under uniaxial impact velocities of 3 m/s, 5 m/s, and 7.8 m/s. The average specific loads increased from 14.83 N·m·g^−1^ to 17.74 N·m·g^−1^ as the impact velocities increased, while the specific peak loads did not exhibit significant impact velocity dependence. Meanwhile, the crushing displacement exhibited the opposite pattern to the average specific load, decreasing with growing impact velocity. Since all specimens were given the same initial kinetic energy and the specimens satisfied the requirement of absorbing the total energy, a higher average specific load necessarily implied a shorter crushing displacement, which could account for the phenomenon in Figure 14. In addition, although the values of the specific loads have been simply normalized to eliminate the effect of specimen density, it can also be found from the figure that the specific peak load value fluctuated in a relatively large range at impact velocities of 5 m/s and 7.8 m/s. This error in data fluctuation mainly came from the following aspects. On the one aspect, the specimens with different densities or even the same density had different pore distributions due to the randomness of the pore structure. Another aspect was the error of the test brought by the influence of the device. Moreover, the data processing method of the specific load derived from Equation (6) had errors. Figure 15 depicts the effect of impact velocity on the contrasted energy absorption and crushing displacement. The results demonstrated that as the impact velocity increased, the specific energy absorption of the specimen increased and the crushing displacement decreased, verifying the inference of Figure 13.

### 3.5. Simulation Results

The uniaxial impact simulation based on finite element analysis was carried out to investigate the impact properties of the specimens and the evolution of in situ collapses within the specimens. The constant initial kinetic energies of 0.6 J and 0.15 J were subjected to the specimens with porosities of 73.0% (0.729 g/cm^3^) and 85.7% (0.386 g/cm^3^), respectively.

**Figure 16:**
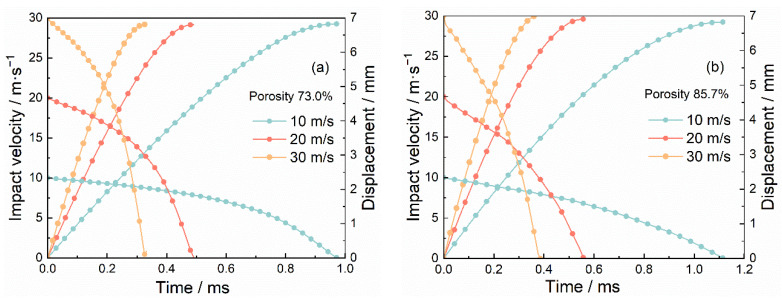
Impact velocity and displacement duration of specimens with (**a**) 73.0% porosity and (**b**) 85.7% porosity at various impact velocities.

**Figure 17:**
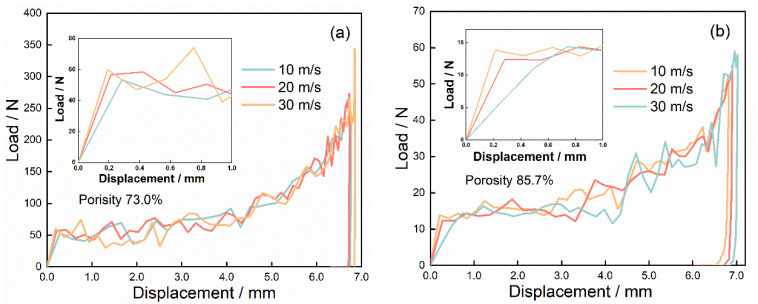
Load–displacement curves of specimen with (**a**) 73.0% porosity and (**b**) 85.7% porosity at various impact velocities.

**Figure 18:**
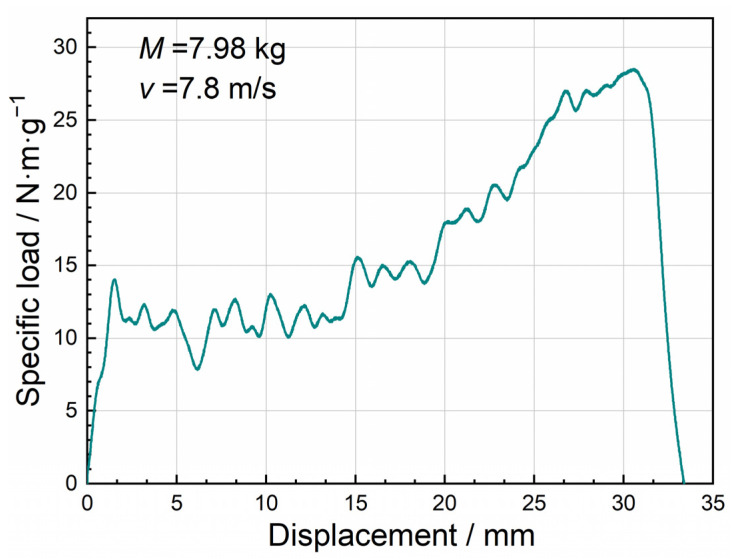
The experimental specific load–displacement curve at velocity of 7.8 m/s.

Figure 16 and Figure 17 depict the impact velocity duration, the displacement duration, and the load–displacement curve of 2D Voronoi-based Al foam specimens with porosities of 73.0%, and 85.7% at various impact velocities of 10 m/s, 20 m/s, and 30 m/s. It can be noticed that the load–displacement curve conformed to the pattern of the typical load–displacement curve of the impact experiment but showed an obvious hardening phenomenon during the second stage. The load of the specimen tended to increase significantly and exceeded *P*_cr_ as the compression displacement exceeded 3 mm, and several experimental impact curves also presented similar characteristics as shown in Figure 18. The peak load and average load of the specimens during the dynamic impact were distinctly lower with the growing porosity. While the impact load of specimens with the same porosity at different impact velocities did not appear significantly different, the corresponding stress and deformation nephograms of the specimens under different impact velocities were also almost identical. This may be associated with the neglect of several material effects, e.g., gas compression effect, inertia effect, and thermal effect. Table 2 captured the critical impact properties of the specimens with porosity of 73.0% and 85.7% during compression at different impact velocities. The peak load of the specimen with a porosity of 73% had a slight increase from 53.88 N to 61.12 N with increasing impact velocity. However, combining the variation of peak loads for two specimens with different porosities at different impact velocities, the peak loads did not present obvious velocity dependence over the range of velocity variation, which was similar to the results in Figure 14. In addition, the specimens with higher porosity (lower density) have a longer crushing displacement at the same impact velocity, matching the inference in Section 3.3. Most of the initial energy from the hammer was absorbed and the requirement for energy absorption of the impact system simulation was satisfied, which indicated that the simulation achieved the expected results.

**Figure 19:**
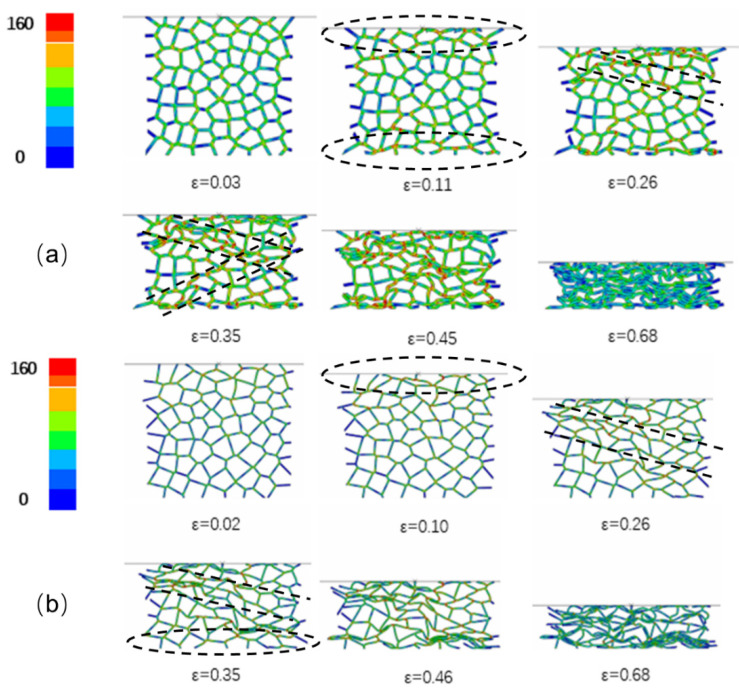
Deformation process of specimens with (**a**) 73.0% porosity and (**b**) 85.7% porosity under uniaxial impact (10 m/s).

**Figure 20:**
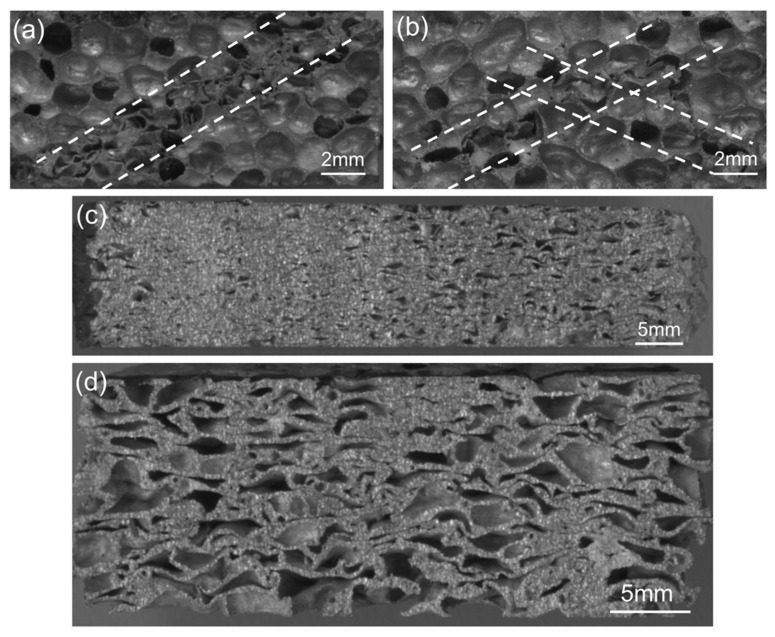
Typical deformation morphologies of specimens during uniaxial impact experiment (**a**) initial deformation band (**b**) X-shaped quasi-conjugate shearing deformation (**c**,**d**) post-impact morphologies of specimens with different densities.

Figure 19 shows the deformation process of the specimens during uniaxial impact (10 m/s), exhibiting the complete in situ collapse evolution of internal foam structure. Mises stress contour plots were employed to indicate the local stress variations within the samples at different compression stages. The legend in the figure depicts the range of Mises stress denoting from blue to red, in ascending order from blue (0 MPa) to red (160 MPa) color. Specimens with porosities of 73.0% and 85.7% demonstrated the slightly different deformation behavior. The impact deformation process of the specimens could be divided into three stages: (1) end deformation; (2) formation of internal deformation band; (3) overall compaction. At the initial stage of impact deformation, the cells near the impact surface of the specimen with 85.7% porosity deformed first, which was consistent with the actual initial compressive deformation under impact observed for the closed-cell Al foam specimen in Figure 8. In contrast, the cells close to the impact and back-impact surfaces of the specimen with 73.0% porosity distorted almost simultaneously. As the strain reached 0.26, the cells that first underwent deformation were almost compacted and the inner cells gradually formed a distinct deformation band. Figure 20a displays the typical deformation band observed during the impact experiment for comparison. The initial deformation band progressively hardened with the proceeding compression, while the cells near the back-impact surface of the specimen with 85.7% porosity also transformed from buckling to compaction. The continuous formation and compaction of multiple deformation bands constituted the main deformation pattern of the specimens in the second stage under the uniaxial impact. Figure 19a (*ε* = 0.35) and Figure 20b show the X-shaped quasi-conjugate shearing deformation. At the end of the impact compression (*ε* = 0.68), almost all the cells within the specimen of 73.0% porosity were compacted, forming a nearly compacted body under the drop hammer. However, the specimen with 85.7% porosity at the same strain still contained numerous cells that were not compacted. Similarly, the different post-impact morphologies for specimens of different densities were frequently observed during the impact experiment, as shown in Figure 20c,d.

## 4. Conclusions

An analytical method for dynamic responses of the cellular structure was developed to evaluate the reliability and reproducibility of uniaxial impact behavior. The dynamic response of closed-cell Al foams was investigated by drop hammer impact test at velocities of 3 m/s, 5 m/s, and 7.8 m/s. From the above-presented analysis, the following important conclusions could be drawn:

(1) The constant initial kinetic energy of 246.84 ± 3.27 J was subjected to the drop hammer impact system to ensure the tested specimen could fully absorb the energy.
(2) During uniaxial impact crushing, specimens with different densities presented three damage modes containing steady state compression, nonsteady state compression, and mixed compression. In addition, the closed-cell Al foam specimens that underwent steady-state compression showed two deformation stages: the initial compression stage and the progressive crushing stage. Four failure modes during uniaxial impact were also found, representing separate energy absorption mechanisms.
(3) The average specific loads increased from 14.83 N·m·g^−1^ to 17.74 N·m·g^−1^ as the impact velocities increased, while the specific peak loads did not exhibit significant impact velocity dependence.
(4) The dynamic-specific energy absorption value was higher than the quasi-static value. Additionally, the specific energy absorption of the specimens increased with the increasing density (0.267 g/cm^3^–0.653 g/cm^3^) and the impact velocity. However, the specimens with higher specific energy absorption seemed not to indicate better cushioning performance due to the shorter crushing displacement.
(5) The uniaxial impact simulations of two-dimensional (2D) Voronoi-based foam specimens with 73.0% and 85.7% porosity were conducted at higher impact velocities of 10 m/s, 20 m/s, and 30 m/s.
(6) The simulation results of impact properties and deformation behavior agreed reasonably well with the experimental results, exhibiting similar velocity insensitivity of peak loads and deformation morphologies during uniaxial impact.
(7) The numerical impact deformation process of closed-cell Al foam specimens could be summarized sequentially as end deformation and internal progressive deformation through the formation of multiple deformation bands.

## Figures and Tables

**Table 1:** Parameters settings for the impact simulation.

Porosity of Specimen	Initial Kinetic Energy (J)	Impact Velocity (m/s)	Mass of Hammer (Kg)	Step Time (s)
73.0%	0.6	10	0.0120	0.0010
73.0%	0.6	20	0.0030	0.0007
73.0%	0.6	30	0.0014	0.0004
85.7%	0.15	10	0.00300	0.0010
85.7%	0.15	20	0.00075	0.0007
85.7%	0.15	30	0.00033	0.0004

**Table 2:** Impact properties of closed-cell Al foam specimens.

Specimen’s Porosity (%)	Impact Velocity (m/s)	Peak Load (N)	Crushing Displacement (mm)	Energy Absorption (J)
73.0	10	53.88	6.77	0.603
73.0	20	56.25	6.73	0.599
73.0	30	61.12	6.86	0.601
85.7	10	13.70	6.82	0.146
85.7	20	12.50	6.90	0.145
85.7	30	14.26	7.02	0.143
